# COVID‐19 and tourism: What can we learn from the past?

**DOI:** 10.1111/twec.13157

**Published:** 2021-06-17

**Authors:** Martina Aronica, Pietro Pizzuto, Caterina Sciortino

**Affiliations:** ^1^ Department of Economics, Business and Statistics (SEAS) University of Palermo Palermo Italy

**Keywords:** COVID‐19, health systems, international arrivals, pandemics, tourism, uncertainty

## Abstract

The impact of the COVID‐19 crisis on tourism flows is without precedent in terms of speed and severity. In this paper, we try to infer a possible future scenario for the tourism sector, evaluating the medium‐term effects of past pandemics on tourist arrivals. We find that pandemics lead to a persistent decline in tourist arrivals, with the effects being larger in developing and emerging countries. Interestingly, the effects are heterogeneous across countries and episodes, and depend on several economic conditions such as the overall health system performance, the severity of the shock, and the uncertainty induced by the pandemic event.

## 1 INTRODUCTION

The COVID‐19 first appeared in China in December 2019 and rapidly spread to the rest of the world, causing one of the deepest economic crises since the Great Depression in the 1930s. The decrease in GDP levels, the contraction of trade, and the increase in unemployment are just some of the immediate effects of this crisis that, unfortunately, is likely to last for a long time (OECD, 2020).

The rapid transmission of the virus and the high number of asymptomatic people induced governments to shut down the activity in many sectors and impose travel and mobility restrictions (Zimmermann et al., 2020). Many countries completely closed their borders to all foreign nationals (e.g. Russia and the U.S.). In contrast, some others closed only partially, conditioning the entrance in their territory on traveller's citizenship or point of origin (e.g. Greece and Austria). Therefore, tourism has been one of the most affected sectors.

Recent data show that international tourist arrivals fell sharply in March 2020, approximately 57 per cent, with a decline between 69 and 84 per cent being recorded for the whole year with respect to 2019 numbers (UNWTO, 2021). Although signs of recovery have been registered in the summer due to an increase in proximity tourism, a remarkable upturn for domestic demand would probably occur in the second half of 2021 (UNWTO, 2020, 2021). Even worse are the forecasts of the International Air Transport Association (IATA, 2020), which suggest that a full recovery of international air travel at the pre‐COVID‐19 levels will take place not before 2023–2024. However, the scenario for global tourism flows will depend, among other things, on the future course of the pandemic and governments' release of travel restrictions.

Given this event's extraordinary dimension, current economic research has mainly focused on analysing the short‐term impact of COVID‐19 and forecasting the future economic panorama (Baker et al., 2020; Bekkers & Koopman, 2020; Li et al., 2020; McKibbin & Fernando, 2020). Following a different perspective, some studies try to infer its possible consequences by analysing past pandemics' economic effects (Barro et al., 2020; Furceri et al., 2020a; Jordà et al., 2020). Indeed, despite the substantial economic impact that COVID‐19 already had, there is a lot of uncertainty on this pandemic's medium and long‐run implications. Therefore, the study of historical events can be a useful guide to understand how the actual pandemic could end up.

Few studies adopt such a broad perspective focusing on the tourism sector (i.e. Gössling et al., 2020) since the existing literature often considers one country and one event (see Novelli et al., 2018 and references cited therein). However, the focus on a single pair “country‐pandemic” has limited use to explain both the social and economic consequences of these kinds of events (see Zenker & Kock, 2020).

To fill this gap, this paper provides evidence on the impact of pandemics and major epidemics (hereinafter “pandemics”) from the past two decades on international tourist arrivals to infer the possible future scenario after the COVID‐19 era. Using data from the World Development Indicators database for a large sample of countries with different degrees of development, this paper investigates whether and how the shocks induced by pandemic events affect international tourism. Our results show that major epidemics and pandemics cause a long‐lasting decline in tourist arrivals. The effects tend to be heterogeneous across countries and episodes and depend on several economic conditions. Notably, we observe a more considerable drop in tourist arrivals in developing and emerging countries and in those countries with a lagging health system. Likewise, more widespread pandemics (like H1N1) tend to hit the tourism sector more than major epidemics confined to specific countries or areas. The effects also depend on the degree of uncertainty induced by the pandemic event and its severity. In this respect, results show that higher uncertainty and severity are associated with the deepest fall in tourist arrivals. These results may help understand the future dynamics of COVID‐19 and provide a useful guide for future policy actions.

The remainder of the paper is organised as follows. Section 2 provides a brief review of the existing literature. Section 3 describes the data and the empirical strategy. Section 4 presents the results. Finally, Section 5 sketches some conclusions and policy implications.

## 2 LITERATURE REVIEW

The COVID‐19 pandemic had and is still having, significant influences at either social, political, or economic levels. The measures put in place to contain the spread of the virus, for example travel bans and quarantines, have strongly hit the tourism sector. Indeed, the tourism industry is heavily conditioned by external events, and tourism flows are highly sensitive to destination countries' health and security conditions (see Blake & Thea Sinclair, 2003; Prideaux et al., 2003).

The tourism sector has experienced several crises not only due to past pandemic events (e.g. SARS, H1N1) but also to natural disasters. An extensive literature has explored the main effects of such events on tourist arrivals (see, among the others, Ma et al., 2020; Novelli et al., 2018; Rosselló et al., 2020; Shi & Li, 2017). The literature on the consequences of natural catastrophes, for example earthquakes and tsunamis, provides evidence of a contraction of tourist flows in the affected area. For instance, Rosselló et al., (2020) using data on both natural disasters and human‐made crises and international tourism flows, show a declining trend of tourist arrivals as a short‐term side effect of these events. In particular, the authors argue that this decline derives from the objective impossibility of the tourism industry to operate (tourism services cannot be delivered) and from tourists' risk perception. Similar results are in Ma, Chiu, et al., (2020). They focus on the impact of earthquakes and terrorist attacks showing that the former exert greater adverse effects on tourism flows. The effects are heterogeneous and depend on the kind of disaster considered and its intensity. They also depend on government responses. Therefore, many authors underline the need for the tourism industry to develop a “disaster planning framework” to accelerate the recovery process (Murphy & Bayley, 1989; Prideaux, 2004).

Besides natural disasters and human‐made crises, various pandemic events have characterised human history, causing adverse shocks for the whole economy and, in particular, for the tourism sector. Indeed, this is not the first time that a coronavirus invests the world community. Recalling the Severe Acute Respiratory Syndrome (SARS, 2003) and the Middle Eastern Respiratory Syndrome (MERS, 2012), the novel coronavirus emergency is the third episode that takes the world off guard.

Numerous studies concentrate on the impact of pandemic episodes on tourism, and many tourism researchers devoted their attention to the need for the authorities to determine quick responses to the ongoing emergency (Jamal & Budke, 2020). Indeed, as highlighted by Zeng et al., (2005), who evaluate the impact of the SARS crisis on the Chinese tourism sector, the tourism industry suffers short‐term negative effects from this kind of crises, but it is usually highly resilient and, thus, able to cope with a rapid recovery if supported by adequate plans. Other studies (i.e. Kuo et al., 2008 and McAleer et al., 2010) compare the impact of SARS and the Avian Flu epidemic on Asian tourist inflows, showing a greater destructive effect of the former on tourists inbound. Mao et al., (2010) concentrate, instead, on the post‐SARS recovery showing that a rapid recovery can occur only with the application of two joint measures: macro‐level strategies, such as mass media campaigns to restore public confidence, and micro‐level strategies like targeted marketing actions, to reduce individuals' risk perception and restore security sensitivity.

Looking at MERS, Joo et al., (2019) find a considerable impact of the induced crisis for the tourism and tourism‐related service sectors in the case of the Republic of Korea. Similarly, Shi and Li (2017) look at MERS' impact on South Korea's tourist arrivals. Particularly, they distinguish different tourist demand types, for example total arrivals, tourist arrivals, and business arrivals, and show that MERS' outbreak strongly affected only total and tourist arrivals from China.

However, coronaviruses are not the unique threat to health conditions and the global economy. Indeed, other notable epidemics/pandemics affected the world in the last two decades: the Swine flu (H1N1) originated in Mexico and rapidly spread worldwide; and the Ebola that in 2014 hit South Africa, causing around 11,000 deaths. Also in these cases, the existing literature has shown the shrinking effects of these epidemic/pandemic episodes on the tourism sector of selected countries. This evidence is corroborated, for example, in Page et al., (2012) who investigate the joint impact of the Swine flu and of the 2008 economic crisis on tourism demand in the U.K. and in Novelli et al., (2018) who analyse the effect of Ebola in the Gambia. Particularly, Novelli et al., (2018) point out that the consequences of these events are even worse in developing countries, which already suffer from inefficient infrastructures and capital for new investments. Remarkably, the adverse effects of health crises may also be associated with a consistent “neighbourhood” effect, as highlighted by Maphanga and Henama (2019). They show how the damaging effects on the tourism sector coming from the outbreak of Ebola in Western Africa were associated with the entire African continent, thus, propagating the crisis even to less affected areas.

As earlier discussed, a growing interest in assessing the impact of the current pandemic is emerging. Although the tourism sector has experienced several crises, none of them had the dimension and the extraordinariness of COVID‐19 that has influenced not only the economic but also the social dimension of tourism. Indeed, apart from mobility restrictions, it has changed travel behaviour, reducing the willingness to take a trip (Zhang et al., 2020), it has increased the desire to move by private means of transport, and it has led people to postpone planned trips of at least 6 months after the restore of safe health conditions (Li et al., 2020).

Current studies have mainly concentrated on analysing the short‐term implications of this pandemic and forecasting future economic panorama. For instance, McKibbin and Fernando (2020) estimate the possible costs of the COVID‐19 outbreak, discussing seven possible future scenarios. Baker et al. (2020) argue that the contraction in GDP levels will be mainly caused by the COVID‐19 induced uncertainty. Zimmermann et al., (2020) suggest a possible relationship between globalisation and pandemics with more globalised countries being affected faster and with a larger impact by COVID‐19. Bekkers and Koopman (2020) investigate the expected effects of the current pandemic on international trade, discussing three possible scenarios. Pahl et al., (2021) try to infer the direct impact and the associated spillover effects that COVID‐19 induced crisis may have in developing countries through Global Value Chains (GVCs).

From a different perspective, some studies try to infer the possible future consequences of COVID‐19, analysing the impact of past pandemic events on global tourism (see Gössling et al., 2020). Indeed, following the COVID‐19 crisis interesting social implications came out for tourism enterprises and employees as well. For example, since a number of workers are in the ‘shadow economy’, there would be the need to emerge in order to benefit from the government's financial supports (Williams, 2020). As shown by Furceri et al., (2020b) pandemic events tend to push people into even more precarious work in the form of self‐employment or in the informal sector. Moreover, the pandemic may affect residents' perceptions of the risks posed by tourism activity. On this point, Qiu et al., (2020) try to estimate the social cost borne by residents in touristic destinations during the COVID‐19 pandemic in three cities of China. In particular, they estimate residents' willingness to pay to reduce the harmful effects of tourism inflows during the pandemic event and show that their willingness was high and, surprisingly, even higher for younger residents.

However, existing literature has often adopted a case‐study approach, with few studies discussing and comparing the different pandemic episodes in a unique framework. Our research contributes to the existing tourism research investigating in a unique framework different past pandemics and countries. Notwithstanding the uniqueness of COVID‐19, a lot can be learned from the past to understand how the current pandemic could end up.

## 3 DATA AND EMPIRICAL STRATEGY

The analysis focuses on the impact of the most important pandemic events of the last two decades: SARS (2003), H1N1 (2009), MERS (2012), Ebola (2014), and Zika (2016). To this end, in line with Ma et al., (2020) and Furceri et al., (2020a), we define a dummy variable (the pandemic event) assuming value 1 when WHO declares a pandemic for the country and 0 otherwise. Data on the number of international tourist arrivals are from the World Bank's World Development Indicators database and cover an unbalanced sample of 183 countries for the period 1995–2018.^1^

Two empirical specifications are used to examine the effect of pandemics on tourist arrivals. The first consists of tracing out the average response of arrivals to major epidemics and pandemics. The second allows this response to vary across countries according to different conditions.

Following the approach proposed by Jordà (2005), in the first specification, we estimate the impulse response functions (IRFs) based on local projections of the effect of pandemics on international tourist arrivals:

(1) $$y_{i,t+k}‐y_{i,t‐1}=\alpha_{i}^{k}+\varphi_{t}^{k}+\beta^{k}D_{i,t}+\theta^{k}X_{i,t}+\varepsilon_{i,t+k}$$

where, $y_{i,t}$ is the log of international tourist arrivals for country *i* in year *t*; $\alpha_{i}$ are country fixed effects to control for the differences in countries’ average tourist arrivals; $\varphi_{t}$ are time fixed effects, included to control for global shocks (e.g. fluctuation in oil prices or the global business cycle); $D_{i,t}$ is a dummy variable indicating a pandemic event affecting country *i* in year *t*. Finally, the vector $X_{i,t}$ includes two lags of the dependent variable and the pandemic dummy.

Specifically, the local projection approach consists of running a sequence of predictive regressions – one for each time horizon – of a variable of interest on a structural shock (in our case, tourist arrivals and pandemics, respectively). The impulse response function is then obtained from the sequence of regression coefficients of the structural shock. Thus, equation (1) is estimated for each horizon (year) *k* = 0, …, 5. Impulse response functions are obtained from the estimated coefficients $\beta^{k}$. Confidence bands are based on robust standard errors clustered at the country level.

This approach is particularly suited to assess the dynamic response of the variable of interest in the aftermath of a shock (Ramey & Zubairy, 2018) and is an alternative way to estimate IRFs without specifying a vector autoregressive model (Autoregressive‐Distributed Lag or ADL). ADL models tend to be sensitive to some misspecifications, such as choosing the number of lags (Teulings & Zubanov, 2014) and long‐lasting effects of shocks may be unduly found, reflecting the use of what Cai and Den Haan (2009) call *one‐type‐of‐shock* models. Instead, the local projection method does not impose the dynamic restrictions embedded in ADL models, and it is particularly suited to estimate non‐linearities in the dynamic response.

Indeed, the second specification exploits the flexibility of the local projection approach in dealing with non‐linearities and state dependency. Specifically, we allow the estimation of the effects of pandemics on tourist arrivals to differ according to some country characteristics (i.e. the severity of the pandemics and the level of uncertainty) when the shock hit. Particularly, we follow the approach adopted by Furceri et al., (2020a), and we extend the baseline specification as follows:

(2) $$y_{i,t+k}‐y_{i,t‐1}=\alpha_{i}^{k}+\varphi_{t}^{k}+F\left(z_{\mathrm{it}}\right)\left[\beta_{L}^{k}D_{i,t}+\theta_{L}^{k}X_{i,t}\right]+\left(1‐F\left(z_{\mathrm{it}}\right)\right)\left[\beta_{H}^{k}D_{i,t}+\theta_{H}^{k}X_{i,t}\right]+\varepsilon_{i,t+k}$$

(3) $$\text{with}\;F\left(z_{\mathrm{it}}\right)=\frac{\exp^{‐\gamma z_{\mathrm{it}}}}{\left(1+\exp^{‐\gamma z_{\mathrm{it}}}\right)}$$

where *z* is, in turn, an indicator of the severity of the pandemic or the uncertainty associated with such exogenous shock, normalised to have zero mean and unit variance. This is a regime‐switching model based on a logistic distribution that controls for the transition from one regime to the other. The weights assigned to each regime vary between 0 and 1 according to the weighting function $F\left(.\right)$, so that $F\left(z_{\mathrm{it}}\right)$can be interpreted as the probability of having a given level of uncertainty (or severity of the pandemic). The parameter γcontrols the smoothness of the transitions from one regime to another with larger values being associated to immediate switches, while smaller ones implying a smoother transition. The coefficient $\beta_{L}^{k}$ is the coefficient in the case of low uncertainty (severity) (that is, when *z* goes to minus infinity) and $\beta_{H}^{k}$ is the coefficient in the case of high uncertainty (severity) (that is when *z* goes to plus infinity).^2^

This approach, firstly proposed by Auerbach and Gorodnichenko (2013), is equivalent to the smooth transition autoregressive model developed by Granger and Terasvirta (1993) and has two main advantages. First, compared to a model in which each dependent variable would be interacted with a measure of economic conditions, it permits a direct test of whether pandemics' effect varies across different regimes. Second, compared to estimating structural vector autoregressions for each regime, it allows the effect of pandemics to change smoothly between regimes by considering a continuum of states to compute the impulse response functions, thus making the response more stable and precise.

## 4 RESULTS

**FIGURE 1:**
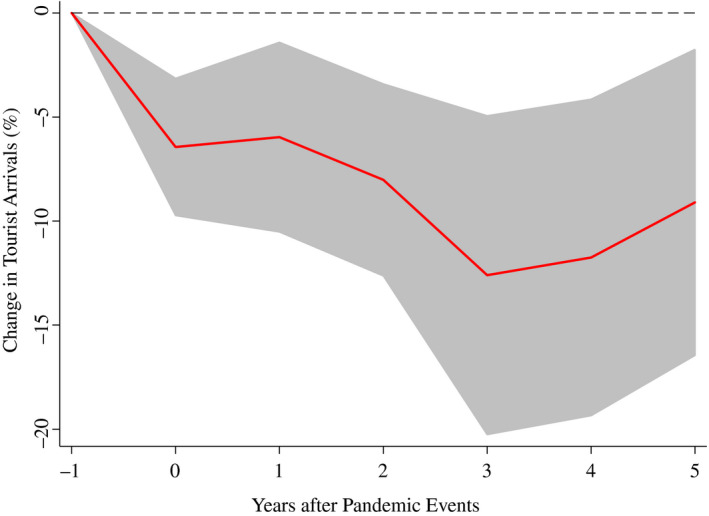
Impact of pandemics on tourist arrivals (%). Note: The chart shows the impulse response functions and the associated 90 per cent confidence bands; *t* = 0 is the year of the pandemic event. Estimates based on equation (1) using a sample of 183 countries over the period 1995–2018

Figure 1 (and Table 1) shows the estimated dynamic response of international tourist arrivals to a pandemic over the 5‐year period following the event, together with the 90 per cent confidence interval. Major pandemics of the latest two decades lead to a long‐lasting decrease in international tourist arrivals, with a peak (average) cumulative fall of about 12.5 per cent 3 years after the event.

**TABLE 1:** Impact of pandemics on tourist arrivals

	*k* = 0	*k* = 1	*k* = 2	*k* = 3	*k* = 4	*k* = 5
*D_i,t_*	−6.438***	−5.968**	−8.018***	−12.60***	−11.75**	−9.107**
	(2.001)	(2.770)	(2.804)	(4.656)	(4.621)	(4.467)
*D_i,t−1_*	−1.265	−2.504	−7.672	−8.848*	−5.818	−3.479
	(1.954)	(2.291)	(5.169)	(5.094)	(4.669)	(5.356)
*D_i,t−2_*	−1.864	−5.622	−8.018**	−7.828**	−6.185	−2.969
	(1.972)	(3.891)	(3.789)	(3.885)	(4.957)	(6.176)
${\Delta y}_{i,t‐1}$	−0.057	−0.098***	−0.171***	−0.169***	−0.195***	−0.270***
	(0.035)	(0.031)	(0.038)	(0.054)	(0.057)	(0.061)
${\Delta y}_{i,t‐2}$	−0.046*	−0.136***	−0.163***	−0.161***	−0.217***	−0.247***
	(0.025)	(0.038)	(0.052)	(0.050)	(0.054)	(0.067)
Observations	3445	3251	3068	2881	2700	2523
*R* ^2^	0.087	0.149	0.218	0.274	0.322	0.387

Note:

Estimates are obtained using a sample of 183 countries over the period 1995–2018 and are based on equation (1). Standard errors in parentheses are clustered at the country level. Country and time fixed effects included but not reported.

*** *p* < 0.01.

** *p* < 0.05.

* *p* < 0.1.

**FIGURE 2:**
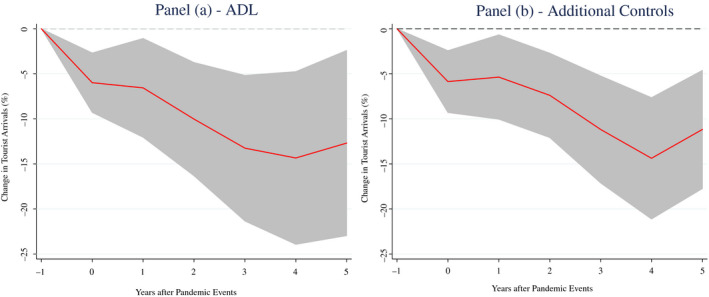
Impact of pandemics on international tourist arrivals (%) – Robustness checks. Note: Impulse response functions are estimated using a sample of 183 countries over the period 1995–2018. The graphs show the response and 90 per cent confidence bands. The *x*‐axis shows years (*k*) after pandemic events; *t* = 0 is the year of the pandemic event. The specification in Panel (b) includes several control variables such as proxies for the level of economic development (log of real GDP), trade openness (imports and exports as a share of GDP), international competitiveness (price level ratio of PPP conversion factor (GDP) to market exchange rate) and population density (people per sq. km of land area).

Interestingly, our results are robust to an alternative methodology (autoregressive distributed lag model ‐ ADL) and to the inclusion of additional control variables in the model (Figure 2 – Panel A and B, respectively).

As indicated by previous studies, the impact of pandemics may be heterogeneous across both countries and episodes (see Furceri et al., 2020a; Ma, Rogers, et al., 2020). For example, less developed countries differ from advanced economies in both the economic structure and the policy instruments that can be used to offset the negative consequences generated by pandemics. In addition, among them, there are several tourism‐dependent economies, that in general, tend to experience more substantial negative economic consequences from travel and mobility restrictions (on this point, see Mooney & Zegarra, 2020).

**FIGURE 3:**
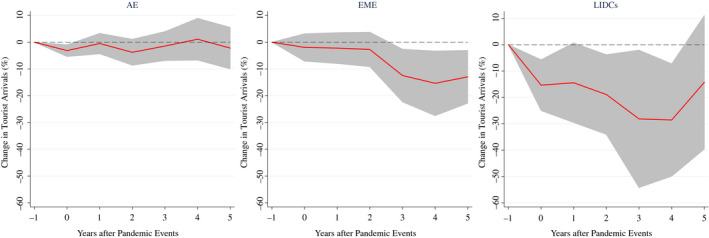
Impact of pandemics on tourist arrivals (%) – by country groups. Note: Impulse response functions are estimated using, in turn, a sample of selected countries (38 AE; 90 EME; 55 LIDCs) over the period 1995–2018. The chart shows the response and 90 per cent confidence bands; *t* = 0 is the year of the pandemic event. Estimates based on equation (1)

To formally test this hypothesis, we re‐estimate equation (1) grouping countries according to the level of development. Particularly, we group countries in Advanced Economies (AE), Emerging Market Economies (EME), and Low Income and Developing countries (LIDCs). Figure 3 shows that, unlike advanced countries, EME and LIDCs seem to be the most affected by pandemics. Notably, the average cumulative decline in tourist arrivals 4 years after the outbreak of a pandemic event is about 12 and 28 per cent, respectively.

Less developed economies also tend to have worse performances in the health system than advanced ones. This appears when looking at both the Global Health Security (GHS) Index – developed in 2019 by a panel of experts from the Johns Hopkins Center for Health Security, the Nuclear Threat Initiative (NTI), and the Economist Intelligence Unit (EIU) – and at the Health Efficiency Index developed by the World Health Organization (WHO) in 2000 (Tandon et al., 2000).

Drawing from the GHS Index, clearly emerges that advanced countries have better scores (average value of about 60). In contrast, emerging economies and low‐income and developing countries show lower levels of the index with average values of about 39 and 33, respectively. The same applies when looking at the results from the WHO's Health Efficiency Index. Advanced countries perform better (average value of 0.89) than emerging economies and low‐income and developing countries that show lower scores (average values of 0.67 and 0.43, respectively).^3^

**FIGURE 4:**
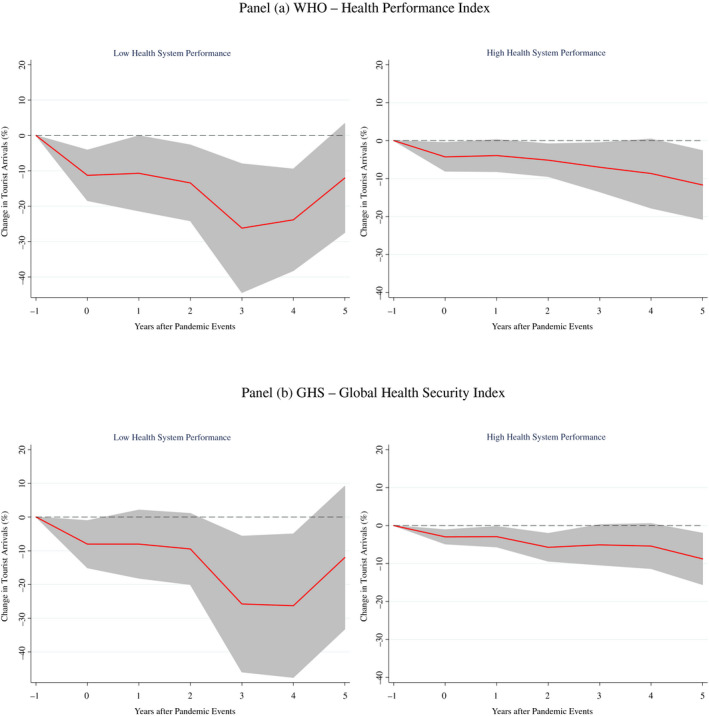
Impact of pandemics on tourist arrivals (%) – by Health System Performance. Note: Impulse response functions are estimated using a sample of 183 countries over the period 1995–2018. The graphs show the response and 90 per cent confidence bands. The *x*‐axis shows years (*k*) after pandemic events; *t* = 0 is the year of the pandemic event

To shed more light on this relationship, we split the sample according to both definitions of health system performance, and we re‐estimate equation (1). The results in Figure 4 show that countries with lower overall health system performances suffer a larger reduction in tourist flows following a pandemic event. The average peak cumulative fall in tourist arrivals is about 27% 3 years after the outbreak of the pandemic event, with the effects being up to three‐four times larger with respect to countries with better health systems.

**FIGURE 5:**
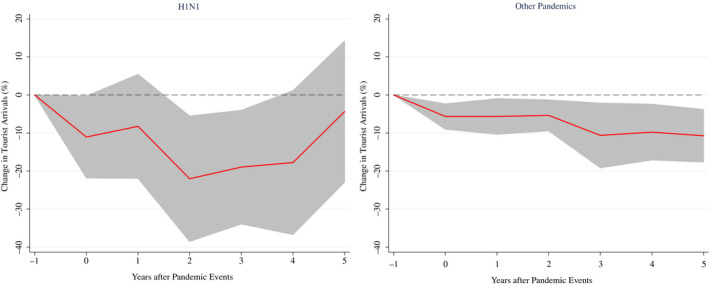
Impact of pandemics on tourist arrivals (%) – by pandemics. Note: The charts show the impulse response functions and the associated 90 per cent confidence bands; *t* = 0 is the year of the pandemic event. Estimates based on equation (1) using a sample of 183 countries over the period 1995–2018

The effects of pandemics may also vary across episodes. Since H1N1 is the most widespread and deadly pandemic in our sample (it affected 158 countries) and it is likely to be the most similar to the COVID‐19 in terms of worldwide spread (even much smaller in scale), we compare the effects generated by this pandemic *vis*‐*à*‐*vis* to those caused by other pandemics in our sample.^4^ Figure 5 shows that the average short‐term fall in tourist arrivals is higher in the case of H1N1 (about −20%) than in other pandemics (about −8%). This is likely due to the diffusion of H1N1 influenza that, indeed, spread around the world, affecting several regions and both inbound and outbound tourism in different areas of the world. Other major epidemics, instead, were mostly confined to specific areas.

**FIGURE 6:**
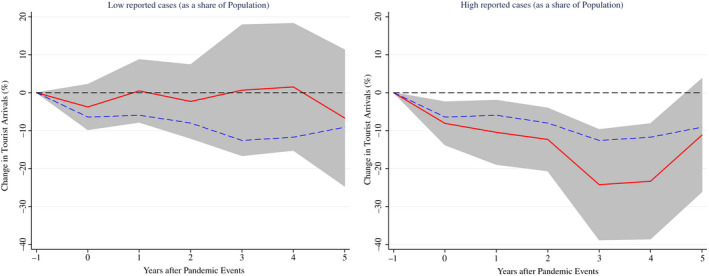
Impact of pandemics on tourist arrivals (%) – The role of the number of cases. Note: Impulse response functions are estimated using a sample of 183 countries over the period 1995–2018. The graph shows the response and 90 per cent confidence bands. The *x*‐axis shows years (*k*) after pandemic events; *t* = 0 is the year of the pandemic event. Estimates based on equation (2) using the ratio of the number of cases to population as a state variable. The dotted blue line denotes the average (unconditional) effect reported in Figure 1. The red lines indicate the estimates for pandemic events associated with very low and high ratio of the number of cases to population

Finally, pandemics may generate heterogeneous effects depending on country‐specific characteristics. Notably, the severity of the shock and the uncertainty associated with the pandemic event may be relevant in shaping its impact on tourist arrivals. To test for these hypotheses, we first estimate equation (2), using the ratio of confirmed cases to population as a state variable.^5^ The results presented in Figure 6 suggest a much stronger decline in tourist arrivals in countries with a higher degree of per‐capita reported cases highlighting the importance of the severity of the pandemic in shaping tourist arrivals' response.

Another channel through which pandemics may generate heterogeneous effects on the tourism sector could be the uncertainty associated with such exogenous shock. The assumption is that the most affected countries (in terms of cases and/or deaths) will be characterised by more uncertainty, and tourists will prefer alternative destinations. To examine the role of uncertainty in assessing the consequences of pandemics on tourist arrivals, we estimate equation (2) using the World Pandemic Uncertainty Index (WPUI). The WPUI is a sub‐index of the World Uncertainty Index (WUI) developed by Ahir et al., (2018). This is constructed by counting the number of times uncertainty is mentioned within a proximity to a word related to pandemics in the Economist Intelligence Unit (EIU) country reports. A higher number means higher uncertainty related to pandemics and vice versa.^6^

**FIGURE 7:**
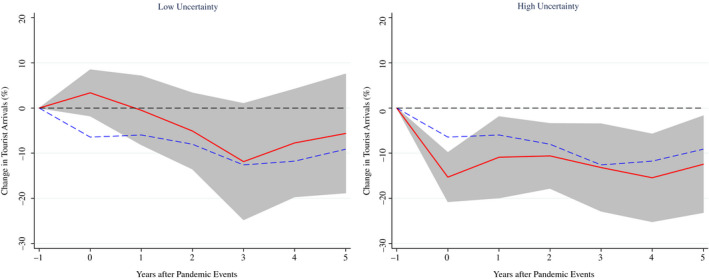
Impact of pandemics on tourist arrivals (%) – The role of uncertainty. Note: The charts show the impulse response functions and the associated 90 per cent confidence bands; *t* = 0 is the year of the pandemic event. Estimates based on equation (2) using a sample of 183 countries over the period 1995–2018. The dotted blue lines denote the average (unconditional) effect reported in Figure 1. The red lines indicate the estimates for pandemic events associated with very low (left panel) and high (right panel) uncertainty

The results in Figure 7 show that the impact of pandemic events on tourist arrivals varies with the uncertainty associated with the shock. In particular, the difference is striking (about 18 percentage points) in the year of the pandemic event (and the first 2 years after the shock) while it attenuates at later stages, even remaining above 7 percentage points. For episodes associated with high uncertainty, the effect is statistically significant and larger than the average effect (the cumulative medium‐term effect on arrivals is about −13 per cent), while it is not statistically significantly different from zero for episodes associated with low uncertainty.

## 5 CONCLUSIONS AND POLICY IMPLICATIONS

The COVID‐19 outbreak has been one of the most impactful and tragic events of modern times, and it is leading to a severe economic crisis, with tourism being one of the most affected sectors. Much is still unknown about the pandemic's future development and its effects on the travelling and hospitality industries. In this paper, we try to infer a possible future scenario for the tourism sector, evaluating the short and medium‐run effects of past pandemics on tourist arrivals. We find that major epidemics and pandemics of the last two decades led to a persistent decline in tourist arrivals, with the effects being larger in developing and emerging countries. Interestingly, the effects are heterogeneous across countries and episodes, and depend on several economic conditions such as the overall health system performance, the severity of the shock, and the uncertainty induced by the pandemic event.

Our findings pose a significant threat to the tourism sector in the post‐COVID‐19 era since the uncertainty around the current pandemic and its severity are much higher than in past outbreaks. The singularity of the current health emergency lies in the geographical coverage of the virus spread and in the massive measures implemented by governments (mobility and travel restrictions, lockdowns, confinements, and so on) to slow down the circulation of the virus. Recent surveys discussed in a report of the Joint Research Center (JRC) of the European Commission (Marques Santos et al., 2020) show that the COVID‐19 health crisis is affecting consumer patterns in the short term, and it may deeply change how people move and choose travel destinations in the medium‐run, causing long‐lasting negative consequences for the tourism sector. Likewise, as discussed in the IMF's 2020 External Sector Report (IMF, 2020), the considerable drop in tourist arrivals will have an outsized impact on countries (such as Costa Rica, Greece, Portugal, Morocco, and Thailand) that heavily rely on foreign travellers—with potentially extensive effects on their economies' national accounts.

Supporting the recovery of the tourism sector is not an easy task for policymakers. Actions should be provided through a combination of demand and supply initiatives to ensure a full, quick, and stable recovery from the COVID‐19 crisis, trying to avoid permanent losses to more exposed economies once the pandemic is controlled. On the demand side, the shift in consumer preferences recorded in the surveys earlier discussed, together with our results from past pandemics suggesting higher fall in tourism flows in the case of higher uncertainty and lower health system performances, advocate policymakers to undertake policies aimed at improving such dimensions. For example, to reduce the uncertainty associated with the pandemic event, tourism authorities could require minimum safety protocols to be observed in restaurants and places related to the travelling and hospitality sectors. Likewise, they could promote and reassure tourists that the destination is safe to attract tourists when COVID‐19 is controlled. To this end, increasing health prevention measures is the basis for maintaining a healthy and sustainable tourism industry. Indeed, the decision‐making process regarding tourism destinations is highly influenced by the perception that people have towards the destination itself (Ma, Chiu, et al., 2020).

On the supply side, instead, there is a need for coordinated policies aimed at preserving productive assets in the short term. Governments should provide low‐interest loans and transfers to companies. They should also ensure full support to people employed in the tourism sector, adopting policies targeted to preserve their human capital and protect workers with temporary contracts that are the most affected by the crisis. These are crucial elements to prepare the ground for the recovery.

Alongside this, the shift in tourists' preferences towards less crowded destinations may foster novel forms of alternative and more sustainable tourism. Policymakers should take this opportunity to increase the diversification of the national offer by promoting interesting places with high potential (i.e. small villages, parks, mountain and protected areas, and rural areas – that is rural and nature tourism) but still not fully recognised as international tourist destinations. This approach can also contribute to local development in less advanced or remote places and help to address a number of adverse effects of mass tourism that were in place before the COVID‐19 outbreak.

Finally, diversifying tourism value chains and making places less tourism‐dependent could be an option to prevent the harmful effects of possible future health crises and to increase the resilience of more vulnerable economies.

## CONFLICT OF INTEREST

The authors declare that they have no conflict of interest.

## Supporting information

Table S1‐S2

## Data Availability

The data that support the findings of this study are available from the corresponding author upon reasonable request.

## Footnotes

^1^
Tables S1 and S2 in the Appendix report the list of countries for each pandemic event and the full list of countries included in the analysis, respectively.

^2^
We use γ = 3.5. $F\left(z_{\mathrm{it}}\right)\;$= 0.5 is the cutoff between low and high economic uncertainty (severity) related to pandemics.

^3^
The WHO index dates back to 2000 and compares all the countries (191) belonging to the WHO, considering different dimensions of the health systems. The index ranges from 0 (lowest performance) to 1 (highest performance). The GHS index, instead, is the first comprehensive assessment of global health security capabilities. It is collected for 195 countries and it is bounded between 0 and 100, with higher values being associated with better health systems. It covers several categories (i.e., prevention, reporting, response, health norms and risks). For further details, see https://www.ghsindex.org/.

^4^
As of September 2020, COVID‐19 infections were confirmed in 235 countries, areas, or territories. https://www.who.int/emergencies/diseases/novel‐coronavirus‐2019 (accessed on September 23, 2020)

^5^
Data sources for number of cases and population are provided in the Appendix.

^6^
The index is the percent of the word "uncertain", and its variants, that appear near the pandemic terms in EIU country reports, multiplied by 1000. Since the index is available on a quarterly basis, to annualize the data we take the sum of quarterly observations. For further details see https://worlduncertaintyindex.com/data/ and https://blogs.imf.org/2020/04/04/global‐uncertainty‐related‐to‐coronavirus‐at‐record‐high/.
