# Supplementary material for: COVID‐19 and tourism: What can we learn from the past?
Source: World Econ. 2021 Jun 17;45(2):430–44. doi: 10.1111/twec.13157 (PMC8242821; doi:10.1111/twec.13157)
Supplement: Supplementary file 1 — Table S1‐S2 [file TWEC-45-430-s001.docx]

**Supplemental Material for the paper**

**“COVID-19 and tourism: What can we learn from the past?”**

**Table S1**. List of Pandemic and Epidemic Episodes

| **Starting year** | **Event Name** | **Affected Countries** | **Number of countries** |
| --- | --- | --- | --- |
| 2003 | SARS | AUS, CAN, CHE, CHN, DEU, ESP, FRA, GBR, HKG, IDN, IND, IRL, ITA, KOR, KWT, MAC, MNG, MYS, NZL, PHL, ROU, RUS, SGP, SWE, THA, USA, VNM, ZAF | 28 |
| 2009 | H1N1 | ­AGO, ALB, ARG, ARM, ATG, AUS, AUT, AZE, BDI, BEL, BGD, BGR, BHR, BHS, BIH, BLR, BLZ, BOL, BRA, BRB, BRN, BTN, BWA, CAN, CHE, CHL, CHN, CIV, CMR, COD, COG, COL, CPV, CRI, CYP, CZE, DEU, DJI, DMA, DNK, DOM, DZA, ECU, EGY, ESP, EST, ETH, FIN, FJI, FRA, FSM, GBR, GHA, GRC, GRD, GTM, GUY, HND, HRV, HTI, HUN, IDN, IND, IRL, IRN, IRQ, ISL, ISR, ITA, JAM, JOR, JPN, KAZ, KEN, KHM, KIR, KNA, KOR, KWT, LAO, LBN, LCA, LKA, LSO, LTU, LUX, LVA, MAR, MDA, MDG, MDV, MEX, MHL, MKD, MLT, MMR, MNE, MNG, MOZ, MUS, MWI, MYS, NAM, NGA, NIC, NLD, NOR, NPL, NZL, OMN, PAK, PAN, PER, PHL, PLW, PNG, POL, PRI, PRT, PRY, ROU, RUS, RWA, SAU, SDN, SGP, SLB, SLV, STP, SUR, SVK, SVN, SWE, SWZ, SYC, SYR, TCD, THA, TJK, TON, TTO, TUN, TUR, TUV, TZA, UGA, UKR, URY, USA, VCT, VEN, VNM, VUT, WSM, YEM, ZAF, ZMB, ZWE. | 158 |
| 2012 | MERS | AUT, CHN, DEU, DZA, EGY, FRA, GBR, GRC, IRN, ITA, JOR, KOR, KWT, LBN, MYS, NLD, OMN, PHL, QAT, SAU, THA, TUN, TUR, USA, YEM. | 25 |
| 2014 | Ebola | ESP, GBR, GIN, ITA, MLI, NGA, SEN, SLE, USA | 9 |
| 2016 | Zika | ARG, ATG, BHS, BLZ, BOL, BRA, BRB, CAN, CHL, COL, CRI, DMA, DOM, ECU, GRD, GTM, GUY, HND, HTI, JAM, KNA, LCA, NIC, PAN, PER, PRI, PRY, SLV, SUR, TTO, URY, USA, VCT, VEN | 34 |
|  |  | **Total Pandemic and Epidemic Events** | **254** |

Note: Based on Ma et al. (2020a)

**Table S2**. List of countries

| **Advanced Economies (AE)** | Croatia | United Arab Emirates |
| --- | --- | --- |
| Australia | Dominica | Uruguay |
| Austria | Dominican Republic | Vanuatu |
| Belgium | Ecuador | Venezuela |
| Canada | Egypt |  |
| Cyprus | El Salvador | **Low Income and** |
| Czech Republic | Fiji | **Developing Countries (LIDCs)** |
| Denmark | Gabon | Bangladesh |
| Estonia | Georgia | Benin |
| Finland | Grenada | Bhutan |
| France | Guatemala | Bolivia |
| Germany | Guyana | Burkina Faso |
| Greece | Hungary | Burundi |
| Hong Kong SAR, China | India | Cambodia |
| Iceland | Indonesia | Cameroon |
| Ireland | Iran | Central African Republic |
| Israel | Iraq | Chad |
| Italy | Jamaica | Comoros |
| Japan | Jordan | Congo |
| Korea | Kazakhstan | Congo, Dem. Rep. |
| Latvia | Kuwait | Cote d'Ivoire |
| Lithuania | Libya | Djibouti |
| Luxembourg | Lebanon | Eritrea |
| Macao SAR, China | Macedonia | Ethiopia |
| Malta | Malaysia | Gambia, The |
| Netherlands | Maldives | Ghana |
| New Zealand | Marshall Islands | Guinea |
| Norway | Mauritius | Guinea-Bissau |
| Portugal | Mexico | Haiti |
| Puerto Rico | Micronesia | Honduras |
| San Marino | Montenegro | Kenya |
| Singapore | Morocco | Kiribati |
| Slovak Republic | Namibia | Kyrgyz Republic |
| Slovenia | Oman | Lao PDR |
| Spain | Pakistan | Lesotho |
| Sweden | Palau | Madagascar |
| Switzerland | Panama | Malawi |
| United Kingdom | Paraguay | Mali |
| United States | Peru | Moldova |
|  | Philippines | Mongolia |
| **Emerging Market** | Poland | Mozambique |
| **Economies (EME)** | Qatar | Myanmar |
| Albania | Romania | Nepal |
| Algeria | Russia | Nicaragua |
| Angola | Samoa | Niger |
| Antigua and Barbuda | Saudi Arabia | Nigeria |
| Argentina | Serbia | Papua New Guinea |
| Armenia | Seychelles | Rwanda |
| Azerbaijan | South Africa | Sao Tome and Principe |
| Bahamas, The | Sri Lanka | Senegal |
| Bahrain | St. Kitts and Nevis | Sierra Leone |
| Barbados | St. Lucia | Solomon Islands |
| Belarus | St. Vincent and the Grenadines | Sudan |
| Belize | Suriname | Tajikistan |
| Bosnia and Herzegovina | Swaziland | Tanzania |
| Botswana | Syrian Arab Republic | Togo |
| Brazil | Thailand | Uganda |
| Brunei | Tonga | Uzbekistan |
| Bulgaria | Trinidad and Tobago | Vietnam |
| Cape Verde | Tunisia | Yemen |
| Chile | Turkey | Zambia |
| China | Turkmenistan | Zimbabwe |
| Colombia | Tuvalu |  |
| Costa Rica | Ukraine |  |

**Data sources for number of cases and population**

**SARS:** <https://www.who.int/csr/sars/country/table2004_04_21/en/>

**H1N1:** <https://en.wikipedia.org/wiki/2009_swine_flu_pandemic_by_country> and <https://www.ecdc.europa.eu/en/seasonal-influenza/2009-influenza-h1n1>

**MERS:** <https://www.ecdc.europa.eu/en/news-events/epidemiological-update-middle-east-respiratory-syndrome-coronavirus-mers-cov-1-0>

**EBOLA:** <https://www.cdc.gov/vhf/ebola/history/2014-2016-outbreak/index.html>

**ZIKA:** <https://www.paho.org/hq/index.php?option=com_content&view=article&id=12390:zika-cumulative-cases&Itemid=42090&lang=en>

**Population**: Data are from the World Bank’s World Development Indicator Database (WDI).
